# Oral steroids for the resolution of otitis media with effusion (OME) in children (OSTRICH): study protocol for a randomised controlled trial

**DOI:** 10.1186/s13063-016-1236-1

**Published:** 2016-03-01

**Authors:** Cherry-Ann Waldron, Emma Thomas-Jones, Rebecca Cannings-John, Kerenza Hood, Colin Powell, Amanda Roberts, Alun Tomkinson, Deborah Fitzsimmons, Micaela Gal, Debbie Harris, Victoria Shepherd, Christopher C Butler, Nicholas Francis

**Affiliations:** South East Wales Trials Unit, Centre for Trials Research, Cardiff University, 7th Floor, Neuadd Meirionnydd, Heath Park, Cardiff, CF14 4XW UK; Centre for Trials Research, College of Biomedical & Life Sciences, Cardiff University, 7th Floor, Neuadd Meirionnydd, Heath Park, Cardiff, CF14 4YS UK; Department of General Paediatrics, Children’s Hospital for Wales, Heath Park, Cardiff, CF14 4XW UK; Cardiff and Vale University Health Board, Child Health Directorate, St David’s Children Centre, Cowbridge Road East, Cardiff, CF11 9XB UK; Ear, Nose and Throat/Head and Neck Department, University Hospital of Wales, Heath Park, Cardiff, CF14 4XN UK; College of Human Health Sciences, Swansea University, Singleton Park, Swansea, SA2 8PP UK; Division of Population Medicine, School of Medicine, Cardiff University, Neuadd Meirionnydd, Heath Park, Cardiff, CF14 4YS UK; The Nuffield Department of Primary Care Health Sciences, University of Oxford, Radcliffe Observatory Quarter, Woodstock Road, Oxford, OX2 6GG UK

**Keywords:** Oral steroids, Otitis media with effusion, Children, Hearing, Randomised controlled trial

## Abstract

**Background:**

Otitis media with effusion (OME) is an accumulation of fluid in the middle ear affecting about 80 % of children by the age of 4 years. While OME usually resolves spontaneously, it can affect speech, behaviour and development. Children with persistent hearing loss associated with OME are usually offered hearing aids or insertion of ventilation tubes through the tympanic membrane. Oral steroids may be a safe and effective treatment for OME, which could be delivered in primary care. Treatment with oral steroids has the potential to benefit large numbers of children and reduce the burden of care on them and on health services. However, previous trials have either been too small with too short a follow-up period, or of too poor quality to give a definite answer.

The aim of the Oral Steroids for the Resolution of Otitis Media with Effusion in Children (OSTRICH) trial is to determine if a short course of oral steroids improves the hearing of children with OME in the short and longer term.

**Methods/design:**

A total of 380 participants (children of 2 to 8 years of age) are recruited from Hospital Ear, Nose and Throat departments in Wales and England. A trained clinician seeks informed consent from parents of children with symptoms for at least 3 months that are attributable to OME and with confirmed bilateral hearing loss at study entry. Participants are randomised to a course of oral steroid or a matched placebo for 1 week. Outcomes include audiometry, tympanometry and otoscopy assessments; symptoms; adverse effects; functional health status; quality of life; resource use; and cost effectiveness. Participants are followed up at 5 weeks, and at 6 and 12 months after the day of randomisation. The primary outcome is audiometry-confirmed satisfactory hearing at 5 weeks.

**Discussion:**

An important evidence gap exists regarding the clinical and cost effectiveness of short courses of oral steroid treatment for OME. Identifying an effective, safe, nonsurgical intervention for OME in children for use in primary care would be of great benefit to children, their families and the NHS.

**Trial registration:**

ISRCTN: ISRCTN49798431 (Registered 7 December 2012)

## Background

Otitis media with effusion (OME) is the most common cause of hearing loss in children in the United Kingdom, and up to 80 % of children are affected by OME by 4 years of age [1]. Overall, the prognosis for OME is good, with more than 50 % of OME episodes resolving spontaneously within 3 months and 95 % within 1 year. However, 30 to 40 % of children have recurrent OME episodes, and 5 % of preschool children (under 5 years of age) have persistent (longer than 3 months) bilateral hearing loss associated with OME [2].

Hearing loss from OME can have an important impact on children’s mood, communication, concentration, learning, socialisation and language development. This may affect other family members and family function. OME in early childhood can affect IQ, behaviour and reading into teenage years [3].

The UK National Institute of Clinical Excellence (NICE) guideline (2008) for OME management recommends a ‘watchful waiting’ period of 3 months, with referral to an Ear, Nose and Throat (ENT) department if hearing is significantly affected, OME persists for longer than 3 months, or if there is suspected language or developmental delay [4]. Treatment options for these children are limited to hearing aids or surgical insertion of ventilation tubes (grommets) through the tympanic membrane. Hearing aids are an effective treatment, but this intervention is not problem-free; children often find them uncomfortable, may feel self-conscious and may become a target for bullying [5].

Although the diagnosis of OME in primary care has increased over the last decade, the number of grommet operations performed in England fell from 43,300 in 1994 to 1995 to 25,442 in 2009 to 2010, primarily as a result of the ‘watchful waiting’ strategy [6]. However, OME remains the most common reason for childhood surgery in the UK and comprises a considerable workload for hospital ENT departments. Furthermore, a wide variation exists in the rate of grommet surgery among regions, which is unlikely to be explained by variation in the disease. In Wales, a six-fold variation exists in the European age-standardised rates of grommet surgery between the highest and the lowest local authorities [7].

Both hearing aids and surgery require referral to secondary care, with major cost consequences. The Department of Health commissioned ‘McKinsey’ report states that the National Health Service (NHS) could save £21 million per year by reducing grommet insertion by a further 90 %, a procedure that they assessed as being ‘relatively ineffective’ [8]. This position has been challenged. Deafness Research UK and the 2009 ENT UK Position Paper conclude that reducing access to grommets will disadvantage thousands of children who have a genuine need of treatment [9, 10].

Antibiotics, topical intranasal steroids, decongestants, antihistamines and mucolytics are all ineffective treatments for OME [11–13]. A rigorous evaluation of anti-inflammatory treatment for OME has been a priority for many years [14]. Cochrane systematic reviews have found insufficient evidence for the effectiveness of both oral steroids and autoinflation (AI) devices in resolving OME in children to recommend implementation but sufficient evidence to recommend further research [12].

A recent trial of an AI device in children with OME and 4 to 11 years of age found a modest effect for some children [15]. However, 80 % of children are affected by OME before the age of 4 years at the time when language development is most rapid, and hearing loss has its greatest effect on language development [3]. Therefore, alternatives to hearing aids or surgery for children less than 4 years of age (who are unable to use an AI device) are required.

Williamson et al. (2009) evaluated topical intranasal steroids for children with OME in general practice and found they are unlikely to be clinically effective for OME [16].

Topical steroids applied through the nose would not be expected to reach the middle ear. However, systemic steroids do reach the middle ear epithelium and modulate OME in animal models [17].

The evidence from in vitro and animal models suggests that steroids reduce effusions and middle ear pressure [18–21]. Various mechanisms have been proposed for a role for steroids in resolving middle ear effusions, including (a) reducing arachidonic acid and associated inflammatory mediators, (b) shrinking peri-eustachian tube lymphoid tissue, (c) enhancing secretion of eustachian tube surfactant with a resultant improvement in tubal function, and (d) reducing middle ear fluid viscosity by its action on mucoproteins [22].

The latest update of the Cochrane review on oral or topical steroids for OME (last search August 2010) found no benefit from intranasal steroids [12]. However, the review did identify evidence of a statistically significant benefit from oral steroids plus antibiotics versus antibiotics alone for OME (5 studies, 409 participants, 23 % in the intervention group and 47 % in the control group with persistent OME at follow-up), and a trend towards a significant benefit for oral steroids versus placebo in the short term (3 studies, 108 participants). Oral antibiotics alone are not effective. The only study to assess the effect of oral steroids on hearing as an outcome was underpowered.

Studies included in the systematic review were short-term, underpowered, often poorly described inclusion criteria and/or did not assess hearing at the time of inclusion, used ears rather than children as the unit of analysis, and used intermediate outcome measures, such as tympanometry results, rather than improved hearing. No cost effectiveness studies of oral steroids for OME were found. Therefore, insufficient evidence exists to recommend oral steroids as a treatment for persistent OME because of inadequate evidence about short-term effect on hearing and cost-effectiveness, and absence of evidence about longer-term effects.

No significant adverse effects of steroids were reported by the studies included in the Cochrane review. However, the number of participants was too small to rule out that possibility. Short courses of prednisolone are widely used in treating children with acute asthma, and adverse events are extremely rare; when they do occur, they are largely limited to behavioural disturbances and dyspepsia and resolve on withdrawal of the steroid drug. The safety of multiple short courses of oral steroid therapy has been evaluated [23]. Short courses of oral steroids such as prednisolone do not have lasting negative effects on bone metabolism, bone density, adrenal gland function or weight or height, even if used on several occasions over the course of a year [24].

An important evidence gap exists regarding the clinical and cost effectiveness of short courses of oral steroid treatment for OME. Identifying an effective, safe, cost-effective, acceptable non-surgical intervention for OME in children (including those in the first 4 years of life) for use in primary care remains an important research priority.

The OSTRICH trial aims to determine the clinical and cost effectiveness of a 7-day course of oral prednisolone (steroid) on improving hearing over the short term in children with bilateral OME, as diagnosed at an ENT outpatient or Paediatric Audiology/Audiovestibular Medicine (AVM) clinic, who have had symptoms attributable to OME present for at least 3 months and current significant hearing loss (demonstrated by audiometry).

## Methods/design

### Objectives

The primary objective of the OSTRICH trial is to determine the clinical and cost effectiveness of a 7-day course of oral steroid on improving hearing at 5 weeks from randomisation in children with bilateral OME, who have had symptoms attributable to OME present for at least 3 months and have current significant hearing loss (demonstrated by audiometry). Oral steroids are likely to have their effect within the first few weeks, and most of the existing evidence is for an effect at 4 to 6 weeks. This is, therefore, the time point at which the maximum effect is expected.

### Design

OSTRICH is a double-blind, individually randomised, placebo-controlled trial involving children with persistent OME and significant hearing loss. It will randomise 380 children (2 to 8 years of age) to receive a 1-week course of oral prednisolone or a matching placebo. All participants are followed up at 5 weeks, 6 months and 12 months after the day of randomisation.

### Setting

This trial is implemented in secondary care sites across Wales and England. Participants are identified in ENT outpatient or Paediatric Audiology and Audiovestibular Medicine (AVM) clinics.

### Participants

Children 2 to 8 years old with symptoms of hearing loss for at least 3 months attributed to OME are eligible to join the trial if they meet the following inclusion criteria.

### Inclusion criteria

The inclusion criteria are as follows:

1. Age 2 to 8 years (for example, has reached the 2^nd^ birthday and has not yet reached the 9^th^ birthday).
2. Symptoms of hearing loss attributable to OME for at least 3 months (or had audiometry proven hearing loss for at least 3 months).
3. Diagnosis of bilateral OME made in an ENT or Paediatric Audiology and AVM clinic on the day of recruitment or during the preceding week.
4. Audiometry confirming hearing loss of more than 20 dBHL averaged within the frequencies of 0.5, 1, 2, and 4 KHz in both ears by pure tone audiometry (PTA) ear-specific insert, visual reinforcement audiometry (VRA) or ear specific play audiometry, or hearing loss of more than 25 dBHL averaged within the frequencies of 0.5, 1, 2, and 4 KHz by sound-field VRA or sound-field performance/play audiometry in the better hearing ear, on the day of recruitment or within the preceding 14 days.
5. First time in the OSTRICH trial.
6. Parent/carer able to understand and give informed consent.

### Exclusion criteria

Children with one of more of the following are not eligible for inclusion:

1. Current involvement in another clinical trial of an investigational medicinal product (CTIMP) or have participated in a CTIMP during the last 4 months.
2. Current systemic infection or ear infection.
3. Cleft palate, Down’s syndrome, diabetes mellitus, Kartagener’s or primary ciliary dyskinesia, renal failure, hypertension or congestive heart failure.
4. Confirmed, major developmental difficulties (for example, are tube fed or have chromosomal abnormalities).
5. Existing known sensory hearing loss.
6. Taken oral steroids in the preceding 4 weeks.
7. Had a live vaccine in the preceding 4 weeks if under 3 years of age.
8. Has a condition that increases their risk of adverse effects from oral steroids (that is, on treatment, the immune system is likely to be modified, or are immunocompromised, such as undergoing cancer treatment).
9. Has been in close contact with someone known or suspected to have Varicella (chicken pox) or active Zoster (shingles) during the 3 weeks prior to recruitment and have no prior history of Varicella infection or immunisation.
10. Already has grommets (ventilation tubes).
11. On a waiting list for grommet surgery and anticipate having surgery within 5 weeks and are unwilling to delay it.

### Trial intervention

Participants randomised to the active treatment arm receive a 7-day course of oral soluble Prednisolone, as a single daily dose of 20 mg for children 2 to 5 years of age or 30 mg for 6- to 8-year olds. The daily dose stated is the most commonly used dose in previous studies of OME and is similar to the standard dose for the treatment of other conditions with inflammatory components (such as asthma). Piramal Healthcare UK Limited has a Medicines and Healthcare Products Regulatory Agency (MHRA) Manufacturing Authorisation and has repackaged and supplied the soluble Prednisolone tablets.

### Control arm

Participants randomised to the control arm receive a 7-day course of oral placebo. This is matched for consistency, colour and solubility, as well as visually, in identical packaging to the active treatment. The placebo is manufactured, packaged and supplied by Piramal Healthcare UK Limited.

The trial medication is prescribed by the patient’s clinician and dispensed by the site Pharmacy.

### Outcomes

The primary outcome is acceptable hearing at 5 weeks from randomisation (4 weeks after conclusion of treatment), where acceptable hearing is defined as ‘less than or equal to 20 decibel hearing level (dBHL)’ averaged within the frequencies of 0.5, 1, 2 and 4 kilohertz (kHz) in at least one ear in children assessed by pure tone audiometry (PTA), ear-specific insert visual reinforcement audiometry (VRA) or ear-specific play audiometry, and ‘less than or equal to 25 dBHL’ averaged within the frequencies of 0.5, 1, 2 and 4 KHz in children assessed by sound-field VRA or sound-field performance/play audiometry. These thresholds are based on national guidelines [25].

### Secondary outcomes

The secondary outcomes assess the longer term (up to 12 months) effects of the intervention on the following:

1. Acceptable hearing at 6 and 12 months (defined as above).
2. Tympanometry (using calibrated standardised tympanometers and modified Jerger classification Types A, B and C).
3. Otoscopic findings.
4. Healthcare consultations related to OME and other resource use.
5. Grommet surgery at 6 and 12 months.
6. Adverse effects.
7. Symptoms (reported by parent and child if appropriate).
8. Functional health status.
9. Health-related quality of life.
10. Short and longer term cost effectiveness.

### Trial procedures

#### Site selection and training

Trial sites are selected on the basis of their recruitment potential and being part of a well-established clinical research network. All sites receive training in trial-specific procedures and good clinical practice (GCP). The training materials are designed specifically to train different staff groups, depending on their roles and responsibilities. For example, the Principal Investigator (PI) and designated trial clinicians are trained on trial-specific tasks including assessing eligibility, taking informed consent and prescribing. OSTRICH nurses (term used in this protocol to refer to clinic nurse, research nurse or clinical studies research officer) are trained in registration, data collection, and handling of the trial medication. Pharmacy staff members are trained in Investigational Medicinal Product (IMP) management procedures, such as storing, allocating and dispensing, as well as temperature monitoring and reconciliation for each randomised patient.

Designated staff members are responsible for cascading training and delegating specific Protocol tasks to other trial site staff.

#### Participant recruitment

**Fig. 1:**
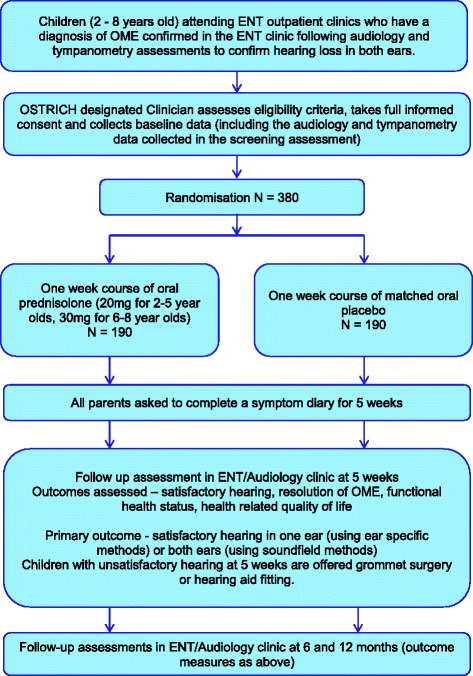
Trial schema and participant flow diagram

The recruitment process is summarised in Fig. 1.

#### Registration and consent

Participating clinicians (ENT or Audiovestibular Medicine) identify eligible patients with bilateral hearing loss and diagnosis of OME during routine outpatient consultations, from current grommet surgery waiting lists or from hearing aid review lists. Additionally, potentially eligible children are identified in Audiology, Audiovestibular (AVM), Paediatric Audiology and Community Audiology clinics, and interested parents/legal guardians will be directed to the participating OSTRICH clinician.

Each child has an audiometry assessment and a clinical assessment (both routine procedures for those attending these clinics) before they are assessed for eligibility to enter the trial. The participating clinician further assesses eligibility, provides the potential participant’s parent/legal guardian with a verbal description of the trial and, if they are interested, provides a comprehensive Participant Information Sheet (PIS). All potential participants’ parents/legal guardians are given sufficient time to read the PIS, ask questions and consider participation before being asked to provide written informed consent if they are willing for their child to participate. Age-appropriate pictorial information sheets are also be provided for children who are old enough to use them.

Parents/legal guardians who consent to take part are asked to sign a consent form, which is also signed by the clinician who is taking consent. Parents/legal guardians are informed that they have the right to withdraw consent from participation in the OSTRICH trial at any time, and that the clinical care of their child is not affected at any time by declining to participate or withdrawing from the trial. Assent may be given by children who are able to understand the age-appropriate information provided and express an opinion regarding their participation.

All participating sites are asked to keep an anonymous screening log of all ineligible and eligible but not consented/not approached patients. These are used to measure potential selection bias.

### Randomisation and blinding

#### Randomisation

Randomisation is coordinated centrally by the South East Wales Trials Unit (SEWTU). The randomisation schedule prepared by the Trial Statistician (TS) comprises random permuted blocks that are stratified by site and child’s age. The IMP Manufacturer (Piramal Healthcare UK Limited) was provided with a list of random allocation numbers linking to either the steroid or placebo. Whether the allocations relate to the steroid or placebo was determined by an independent statistician to ensure the TS remains blinded. The allocation numbers are used to label the trial medication packs. Each trial medication pack has a unique identification number (Trial Pack number).

As children are recruited, they are assigned the next vacant Participant Identification number (PID). Trial medication packs are only released once informed consent has been obtained and a consent form signed. Participants are randomised to receive either the steroid or the matching placebo by receiving the next sequentially numbered Trial Pack allocated to the participant by the site Pharmacy. A designated member of the OSTRICH trial site team (where possible) or the participant’s parent/legal guardian collects the pack from Pharmacy on behalf of the participant. Participant randomisation is considered to have occurred once a consent form is signed and the Trial Pack opened. The Trial Pack number is then entered onto the participant’s case report form (CRF).

#### Blinding

Participants, parent/legal guardians, clinic staff and members of the OSTRICH trial team remain blind to treatment allocation. The unique identification number on each Trial Pack is linked to the randomisation schedule. If clinicians at sites are providing clinical treatment for a serious adverse event (SAE), they contact SEWTU if unblinding is required.

### Data collection

#### Baseline/clinical assessments

An eligibility case report form (CRF) is completed for all consented participants to ensure that they meet the eligibility criteria specified in the trial protocol. The clinician signs the form to confirm that the participant is eligible to be enrolled into the trial.

A participant registration form is completed by the OSTRICH nurse to register the participant and their parent/legal guardian to the trial (this includes collecting names and addresses of the participants and their parent/legal guardians).

The OSTRICH nurse and clinician complete the CRFs, recording medical history and audiometry, tympanometry and otoscopy measurements. Table 1 provides an explanation of the clinical assessments of this trial.

**Table 1:** Clinical measurements

Measurement	Outcome
Audiometry	Hearing in each ear by pure tone audiometry, ear-specific insert visual reinforcement audiometry (VRA) or ear-specific play audiometry) or in both ears together by sound-field VRA or sound-field performance/play audiometry)
Tympanometry (using calibrated standardised tympanometers and modified Jerger classification Type B and C considered abnormal)	Presence of middle ear effusion in each ear
Otoscopy	Appearance of tympanic membrane

In current practice, the recommended standard methods to assess hearing thresholds are ear-specific pure tone audiometry (PTA) at 0.5, 1, 2 and 4 kHz in children 3 years or older, and sound-field visual reinforcement audiometry (VRA) in children under 3. However, equally, those children under 3 years of age may comply with PTA. Therefore, we recommend that the audiologist/clinician use their judgement on the most appropriate method of assessment for the child and, where possible, maintain that method for subsequent follow-ups.

We are aware that ear-specific VRA through the use of insert earphones is considered ‘gold standard’ practice but believe that sound-field VRA will provide a reasonable assessment of the child’s level of hearing and will ensure the feasibility and wider applicability of the trial in a range of research sites due to wider availability.

#### Functional health status and quality of life

The parent/legal guardian is asked to complete a questionnaire booklet comprising the Otitis Media (OM8-30) questionnaire to assess the child’s Functional Health Status [26], the Paediatric Quality of Life Inventory (PedsQL) to measure health-related quality of life [27], and the Health Utilities Index Mark 3 (HUI 3) to measure health utilities [28]. If appropriate, a child’s version of the questionnaire booklet is also completed by the participant, comprising the child self-report version of PedsQL.

#### Diary

The OSTRICH nurse provides parents/legal guardians with a symptom diary to take home and complete over the first 5 weeks. In the first week, this is completed daily to record treatment adherence. Thereafter, it is completed weekly for 4 weeks to record symptoms, adverse events, healthcare resource usage (which includes general practitioner (GP) and practice nurse consultations, procedures, investigations, hospital appointments, accident and emergency (A&E) attendances and any hospital inpatient admissions), additional medication taken, time off school/nursery and parental time off work.

The OSTRICH nurse provides the parent/legal guardian with the trial medication (where possible) or the prescription for the parent/legal guardian to take to the Pharmacy themselves, and discusses taking the trial medication if appropriately qualified to do so. A medication guidance and instructions for use leaflet is also provided to parent/legal guardian. The next clinic appointment (at week 5) is made for the participant to attend, and the parent/legal guardian is advised that there are further follow-up clinic visits at 6 and 12 months.

#### Follow-up assessments

Follow-up assessments for all participants are conducted at week 5 (4 weeks post completion of treatment, + 2-week window), 6 and 12 months (±2-week window).

At the 5-week follow-up appointment, any unused trial medication is collected and returned to the Pharmacy for disposal, and the OSTRICH nurse collects the completed symptom diary from the parent/legal guardian.

Completion of the questionnaire booklets and the clinical assessments (for example, audiometry, tympanometry, and otoscopy) are repeated at each of the follow-up clinic appointments. Although the follow-up of participants is continued for 12 months, after the 5 week assessment all participants resume ‘usual care’, and all treatment decisions are made by their parents in consultation with their clinician.

The questionnaire booklets at 6 and 12 months also include healthcare resource usage to assess use of NHS resources, additional medication taken, time off school/nursery and parental time off work.

In the event that participants’ follow-up appointments are missed at the proposed time points, SEWTU coordinate with the trial site staff, and the parent/legal guardian of the participant is contacted by telephone to rearrange the visit as soon as possible. In the event that telephone contact is not successful, visit reminder letters are sent to rearrange the appointment.

If parents/legal guardians are unable or unwilling to attend a follow-up appointment, they are asked if they are willing to complete the questionnaire booklet that would be sent to them in the post. A freepost self-addressed envelope is provided for parents to return their diary, unused medication and/or questionnaire booklet. Alternatively, they will be given the option of answering a brief questionnaire over the telephone, comprising questions extracted from the diary regarding symptoms (for the 5-week follow-up, if not completing/returning the diary) and quality of life (if not completing and returning a questionnaire booklet).

### Analysis

#### Sample size

The sample size calculation is based on demonstrating a change in the rate of resolution of hearing loss at 5 weeks post randomisation (4 weeks post completion of treatment) from 20 % in the control group to 35 % in the intervention group. OME resolves spontaneously in a high proportion of children, and some studies have found a significantly higher rate of spontaneous resolution. For example, Williamson et al. found a resolution rate in their control group of 47 % [16]. However, we anticipate a lower spontaneous rate of resolution because we will only include children who have been symptomatic for at least 3 months and are recruiting children in a secondary care setting, where a more severe spectrum of illness can be anticipated. The Cochrane review of steroids for OME reported a ratio of proportions for resolution of OME at 2 weeks of 3.80 (95 % CI = 0.93 to 15.52). In the five studies in the Cochrane review of oral steroids versus placebo, overall there was a 23 % recovery rate in the placebo plus antibiotic group and a 47 % recovery rate in the oral steroid plus antibiotic group, which represents a 24 % difference (antibiotics on their own are ineffective) [12]. We have selected a conservative estimate of 1.75 for our effect size (ratio of proportions) because we believe that a 15 % absolute increase in the rate of resolution at 5 weeks would represent a clinically meaningful benefit that could result in a meaningful reduction in unnecessary operations and a related saving in cost for the NHS. In order to demonstrate a difference between 20 % and 35 % with an alpha of 0.05 and 80 % power, we will need 302 participants (nQuery software version 4.0). We will recruit 380 to allow for a 20 % loss to follow-up at 1 year. Although our primary outcome data will be gathered at 5 weeks, we believe that it is important to be able to assess long-term outcomes and, therefore, want to ensure that we will have sufficient power for longer term follow-up assessments.

#### Statistical analysis

The primary analyses will be intention to treat and will employ a logistic regression model to investigate differences in the proportion of children with acceptable hearing at the 5 weeks post randomisation follow-up appointment between the two treatment arms, adjusting for site. Comparisons will be presented as the absolute difference in proportions and the adjusted odds ratio, with 95 % confidence interval and *P* value.

Secondary outcomes with a binary outcome (present/absent), such as satisfactory hearing and presence of effusion, will employ repeated measures logistic regression to investigate differences between the trial arms and over time (5 weeks and 6-month and 12-month follow-ups). Changes in hearing over time will also be analysed to identify children who show early resolution but then relapse. For continuous secondary outcomes, such as PedsQL, and OM8-30 scores, repeated measures linear regression models (using transformations as necessary) will be used to investigate differences between the trial arms and over time (5 weeks, 6 months and 12 months) adjusting for baseline. The duration between the start and the resolution of the symptoms will be calculated and the median (interquartile range) for each randomised group will be presented. A Cox regression model will be used to test whether time to resolution differs between the randomised groups.

A number of outcomes will be calculated from the parents’ diary for the first 5 weeks, such as total time off school/nursery and work (days) and the number of healthcare consultations. These will be analysed using Poisson regression to investigate differences between the two trial arms.

Previous researchers have mapped OM8-30 scores to utility values on the HUI-3 scale. As this trial measures both OM8-30 and HUI-3, it provides the opportunity to evaluate the generalisability of the existing mapping. This will be done by correlating the mapped utility values on the HUI-3 scale (obtained via the mapping formula from the OM8-30 facet scores) with the newly acquired HUI-3 scores.

Possible confounders such as the child’s age and history of atopy and relevant interaction terms will be entered into the primary regression analyses for each of the outcomes in order to conduct pre-specified subgroup analyses. These subgroups will be defined in advance of any analysis based on the best available evidence. Since the trial is powered to detect overall differences between the groups rather than interactions of this kind, the results of these exploratory analyses will be presented using confidence intervals as well as *P* values. No interim analyses are planned.

Full description of the methods to be used will be stated in a trial statistical analysis plan.

#### Economic analysis/cost effectiveness analysis

The cost-effectiveness analysis will be conducted from the perspective of the UK NHS and Personal Social Services and also will consider a broader partial societal perspective, encompassing impacts on patients and their families. The cost-effectiveness component will have two time durations: a within-trial assessment and a longer term time horizon based on decision-analytic modelling and populated from parameter estimates derived from the trial and from information from literature sources relating to long-term effects of hearing difficulties in children. These time periods offer a longer duration than previous studies and will be used, alongside other sources, to arrive at more meaningful estimates of cost-effectiveness.

The costs of the course of oral steroids will be calculated and combined with differences in costs between intervention and control groups to determine overall costs associated with the intervention. The resource utilisation of both groups (consultations, medications, operations, equipment, etcetera) and treatments associated with adverse events, will be assessed through the completion of self-completed questionnaires at baseline, at 5 weeks, 6 months and 12 months and translated into costs using appropriate published unit costs [29].

The difference in overall costs between groups will be compared with differences in outcomes, as specified above, and including quality-adjusted life years (QALYs). QALYs will be computed from PedsQL and HUI 3 and from utilities derived from mapping responses to the OM8-30 questionnaire [30].

A series of one-way sensitivity analyses will be conducted to assess the impact of parameter variation on baseline estimates of the cost-effectiveness ratios and a probabilistic sensitivity analysis undertaken to determine the extent to which the intervention can be regarded as representing value for money.

### Ethical and governance approval

This trial protocol was reviewed and approved by Wales Research Ethics Committee (REC) 3, which is recognised by the United Kingdom Ethics Committee Authority (UKECA). All hospital sites received Research and Development approval from the respective NHS Health Boards and Trusts in Wales and England. These were Cardiff and Vale University Health Board, Aneurin Bevan University Health Board, Cwm Taf Health Board, Abertawe Bro Morgannwg University Health Board, Hywel Dda University Health Board, Betsi Cadwaladr University Health Board, The Royal Wolverhampton NHS Trust, The Newcastle Upon Tyne NHS Foundation Trust, Bradford Teaching Hospitals NHS Foundation Trust, London North West Healthcare NHS Trust, Maidstone and Tunbridge Wells NHS Trust, Royal Free London NHS Foundation Trust, University College London Hospitals NHS Foundation Trust, University Hospitals of North Midlands NHS Trust, Isle of Wight NHS Trust, Sheffield Children’s NHS Foundation Trust, Central Manchester University Hospitals NHS Foundation Trust, Brighton and Sussex University Hospitals NHS Trust, Surrey and Sussex Healthcare NHS Trust, Blackpool Teaching Hospitals NHS Foundation Trust, Walsall Healthcare NHS Trust and Worcestershire Acute Hospitals NHS Trust. Clinical Trial Authorisation was obtained from the Medicines and Healthcare products Regulatory Agency (MHRA).

## Discussion

OSTRICH will be the first adequately powered trial to evaluate the clinical and cost-effectiveness of a short course of oral steroids for the resolution of OME in children in the short term and longer term. Some clinicians already prescribe oral steroids for OME with others opposed to such practice. The analysis proposed by OSTRICH will generate much needed evidence that will greater inform clinicians and will dispel the strongly opposing views about the use of oral steroids for the treatment of OME.

If shown to be effective, the use of oral steroids for OME could benefit children and provide the option of primary care treatment as opposed to costly secondary care.

The economic analysis will assess the cost of any benefits achieved through the use of oral steroids for the resolution of OME in children. In addition to direct costs, this will include any savings (or additional costs) that result from prescribing the oral steroid. For example, costs for the NHS may be lowered if the number of grommet operations that are needed is reduced. This will provide additional important information to healthcare providers and funders to aid them in making the most efficient use of their finite resources.

Conversely, if shown not to be clinically effective, then that information will provide evidence to change practice where they are currently being used, and research efforts could focus on developing alternative pathways for improving the management of OME in children.

The OSTRICH trial will address the important evidence gap regarding clinical and cost effectiveness of short courses of oral steroid treatment for OME. Identifying an effective, safe, cost-effective, acceptable non-surgical intervention for OME in children (including those in the first 4 years of life) for use in primary care remains an important research priority and would be of great benefit to children, their families and the NHS.

## Abbreviations

A&E: accident and emergency
AI: autoinflation
AVM: audiovestibular medicine
AE: adverse event
CRF: case report form
CTIMP: Clinical Trial of Investigational Medicinal Product
dBHL: decibels hearing level
ENT: ear, nose and throat
GCP: good clinical practice
GP: general practitioner
HUI3: Health Utilities Index Mark 3
IMP: Investigational Medicinal Product
KH: kilohertz
NHS: National Health Service
NICE: National Institute for Clinical Excellence
NIHR HTA: National Institute for Health Research Health Technology Assessment
OME: otitis media with effusion
OM8-30: Otitis Media with Effusion Questionnaire
PEDS-QL: Paediatric Quality of Life Inventory
PID: patient identification number
PIS: patient information sheet
PTA: pure tone audiometry
QALY: quality-adjusted life years
REC: research ethics committee
SAE: serious adverse event
SEWTU: South East Wales Trials Unit
TS: trial statistician
VRA: visual reinforcement assessment
UKECA: United Kingdom Ethics Committee Authority
